# Preclinical evaluation of cationic DOTA-triarginine-lipid conjugates for theranostic liquid brachytherapy

**DOI:** 10.7150/ntno.44562

**Published:** 2020-04-22

**Authors:** Wenbo Wang, Frederikke P. Fliedner, Anders E. Hansen, Rasmus Eliasen, Fredrik Melander, Andreas Kjaer, Thomas L. Andresen, Andreas I. Jensen, Jonas R. Henriksen

**Affiliations:** 1 Department of Health Technology, Technical University of Denmark, Produktionstorvet Building 423, DK 2800 Lyngby, Denmark; 2 Rigshospitalet and University of Copenhagen, Dept. of Clinical Physiology, Nuclear Medicine & PET, Cluster for Molecular Imaging, 2100 Copenhagen, Denmark; 3 The Hevesy Laboratory, Department of Health Technology, Technical University of Denmark, Frederiksborgvej 399, DK, 4000 Roskilde, Denmark; 4 Center for Nanomedicine and Theranostics, Technical University of Denmark, 2800 Lyngby, Denmark

**Keywords:** brachytherapy, radiotherapy, cancer, intratumoral, micelles

## Abstract

Liquid brachytherapy is an emerging technology for internal radiation therapy where liquids containing radionuclides are administered directly into solid tumors. These technologies are less invasive than conventional brachytherapy, and can potentially improve the dose coverage and homogeneity of the radioactivity distribution within the tumor. For this purpose, we have developed a novel cationic micelle system for delivery of a range of radionuclides. The system is applicable for emitters of alpha, beta or photon radiation, and enables dose-mapping via theranostic nuclear imaging.

**Methods:** The cationic micelles were developed as linear surfactants comprising the chelator DOTA, a triarginine sequence and a palmitoyl or stearoyl fatty acid chain. The critical micelle concentration of the surfactants was determined, and the micelles were radiolabelled with ^64^Cu or ^177^Lu in high radiochemical purity (>95%). The tumor retention and biodistribution of the ^64^Cu-radiolabeled surfactants, administered as micelles or formulated in liposomes, were investigated *in vivo* by PET/CT in a tumor bearing mouse model.

**Results:** The interaction of the micelles with anionic lipid membranes was demonstrated to be favourable, using a liposome partition assay. *In vivo*, the surfactants formulated both as cationic micelles and liposomes displayed the best intratumoral retention, with micelles providing more homogeneous activity distribution.

**Conclusion:** A cationic, surfactant-based drug delivery system was developed and demonstrated promise as a vehicle for liquid brachytherapy when formulated as micelles or in liposomes. The system enables accurate dosimetry due to the flexible radiochemistry of DOTA.

## Introduction

In brachytherapy (BT), locally applied radioactive materials are administered as part of cancer therapy 1. In conventional BT, sealed radioactive sources are placed in or near the cancerous tissue in order to deliver a local radiation dose with reduced exposure of the surrounding healthy tissue. In clinical applications, BT is often used in combination with external beam radiotherapy (EBRT) for the treatment of solid cancers like cervical, prostate, breast and skin cancer 2, 3. Currently, BT is provided by millimetre sized point sources, which unfortunately results in heterogeneous dose coverage of the tumor 4. Two forms of BT are commonly used, high dose-rate (HDR-BT) and low dose-rate (LDR-BT). In HDR-BT, radioactive point sources containing radionuclides such as iridium-192 and cobalt-60 are temporarily placed in or close to the tumor, providing dose rates of >12 Gy/h 5, 6. In LDR-BT, radioactive sources are permanently implanted and radiation doses are delivered at a lower dose rate (0.4-2 Gy/h). Commonly used radionuclides in LDR-BT are palladium-103 (Main photons: 20-23 keV, 77%) and iodine-125 (Main photons: 27-35 keV, 143%) 7. Both forms of BT have the advantage of providing a high radiation dose to the tumor with minimal exposure of healthy tissues, and the disadvantage of heterogeneous tumor dose coverage and patient discomfort during intratumoral placement of multiple BT sources. For large tumors with ill-defined margins, the entire tumor may not be irradiated efficiently with currently available systems, resulting in poor tumor control 8. Further, current technologies use metal-based encapsulation that absorbs non-photon emissions, hindering the use of alpha- and beta-particle emitting therapeutic radionuclides.

Liquid BT (LBT) is largely experimental and involves the injection of liquids containing radioactive materials directly into the tumor. In LBT, thin needles can therefore be used, and the number of injections is reduced, since the materials spread out in the tissue, making it less invasive. New drug delivery systems for LBT that efficiently distribute and retain radioactive materials in the intended tumor region or sub volume is therefore of significant interest. Such systems can be used in a number of clinical settings, notably (1) as a stand-alone intratumoral injection, (2) as a general boost to EBRT, and (3) as an adjuvant booster therapy to target tumor zones not easily accessible with EBRT. In this latter case, boosts to specific tumor regions or sub volumes is of clinical interest to improve the therapeutic outcome of EBRT, which due to off target toxicity is not feasible by EBRT alone 4. Advanced dosimetry calculations are employed in EBRT to secure accurate and sufficient dosing to cancerous tissue. For this reason, BT systems where the intratumoral dose distribution can be mapped and incorporated in the dosimetry planning for EBRT is highly attractive. Here we present a novel theranostic LBT system that minimizes patient discomfort, as it can be injected through small gauge needles. The system allows for accurate injection and intratumoral irradiation of sub volumes insufficiently covered by EBRT or with known radio-resistance e.g. hypoxic regions. In addition, the delivery system is designed for controlled distribution and increased retention of radionuclides in the tumor, and allows for accurate dose mapping via e.g. positron emission tomography (PET) imaging. To achieve this, we synthesized two novel chelator-triarginine-lipid constructs based on 1,4,7,10-tetraazacyclododecane-1,4,7,10-tetraacetic acid (DOTA). These constructs allow for stable chelation of several theranostic radionuclides and pairs for both PET imaging and brachytherapy, such as ^64^Cu/^67^Cu, ^177^Lu, ^86^Y/^90^Y, and ^68^Ga 9-11.

**Figure 1:**
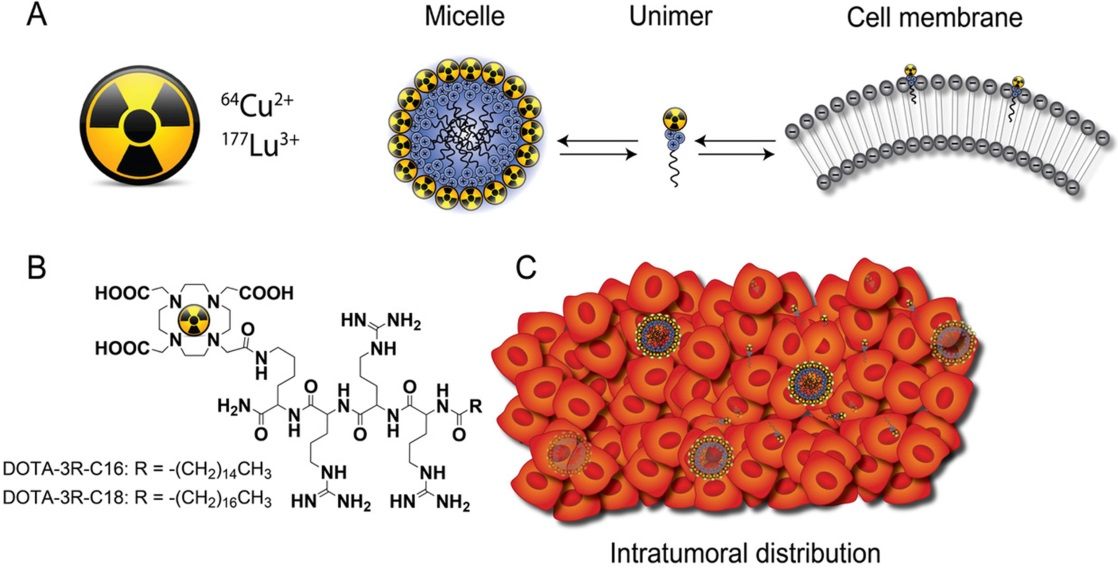
**General concept. (A)** Schematic illustration of the equilibrium of radiolabeled cationic unimers with the cell membrane and the micelles themselves. **(B)** Chemical structures of D3R-C16 and D3R-C18. **(C)** Upon intratumoral injection, the micelles are distributed within the tumor interstitium and single unimers insert into the tumor cell membranes.

Only few new biomaterials attempting to provide both better distribution and retention of injectable radioactive source have been described. For example, the distribution of ^103^Pd-labeled gold-coated nanoparticles was found to be more uniform than conventional metal seeds within the tumor 12, and liposomes radiolabeled with ^186^Re/^188^Re were evaluated as a potential approach in brachytherapy 13. The latter could diffuse throughout the tumor and had higher intratumoral retention than small molecules. However, the distribution of both nanoparticle types was still heterogeneous, making it problematic to correlate therapeutic efficacy with the injected dose. The current DOTA-triarginine-lipid constructs form nanometre size micelles, with an affinity for negatively charged cell membranes, due to the inclusion of cationic arginines (**Figure 1**). These are capable of delivering various radionuclides to the tumor via diffusion through the interstitium. In this process, micelles may disassemble into single surfactant molecules (unimers) that can partition into the tumor cell membranes and deliver a lethal radiation dose. The present work includes a physicochemical characterization of the DOTA-triarginine-lipid constructs and testing using the diagnostic ^64^Cu and therapeutic ^177^Lu radionuclides. The biodistribution and tumor retention of ^64^Cu-labeled DOTA-triarginine-lipid constructs formulated as micelles or liposomes were investigated upon intratumoral administration using PET imaging in mice.

## Materials and Methods

### Materials

9-Fluorenylmethoxycarbonyl (Fmoc) amino acids and *O*-(7-azabenzotriazol-1-yl)-1,1,3,3-tetramethyluronium hexafluorophosphate (HATU) were purchased from GL Biochem or Iris Biotech GmbH. Novasyn TGR resin was purchased from Merck Chemicals GmbH. DOTA-tris(tBu)ester was purchased from Chematech. The freeze dried, premixed stealth liposome mixture of hydrogenated soy phosphatidylcholine (HSPC), cholesterol (CHOL) and 1,2-distearoyl-sn-glycero-3-phospho-ethanolamine-N-[methoxy(polyethyleneglycol)-2000] (DSPE-PEG2000) (565:382:53, molar ratio) was purchased from Lipoid. 1-palmitoyl-2-oleoyl-sn-glycero-3-phospho-(1-rac-glycerol) (POPG) and 1-palmitoyl-2-oleoyl-sn-glycero-3-phosphocholine (POPC) were purchased from Avanti Polar Lipids. Water was collected from a Milli-Q system (Millipore). All other chemicals were purchased from Sigma Aldrich. All the chemicals and reagents were of analytical grade and used without further purification.

Isotonic buffers, containing 10 mM 4-(2-hydroxyethyl)piperazine-1-ethanesulfonic acid (HEPES) with 150 mM sodium chloride (ISO-HEPES-NaCl) or 300 mM sucrose (ISO-HEPES-Sucrose) were prepared and adjusted to final pH values of 7.4.

CT26 (murine colon carcinoma) was purchased from ATTC (Rockville) and cultured in DMEM medium supplemented with 10% fetal calf serum and pen-strep (Invitrogen Inc) at 37 °C and 5% CO_2_ in a humidified atmosphere. A commercial MTS assay was used to assess cytotoxicity. (Promega, CellTiter 96® AQueous One Solution Cell Proliferation Assay).

All radio-TLC analyses were performed on silica gel 60 F254 plates (Merck). 5% (w/v) ammonium acetate (NH_4_OAc) in water-methanol (1:3) was used as eluent for ^64^Cu-D3R-C16 and ^64^Cu-D3R-C18, and 5% (w/v) ammonium acetate (NH_4_OAc) in water- methanol (1:1) was used as eluent for ^64^Cu-DOTA,^ 64^Cu-EDTA, ^177^Lu-D3R-C16 and ^177^Lu-D3R-C18. A MiniGita Star with a Beta Detector GMC probe (Perkin-Elmer) was used for analysis of radio-TLCs with radioactive peaks integrated using associated computer software. All radioactivities were measured either on a Veenstra Instruments dose calibrator VDC-505, or by liquid scintillation counting on a 300 SL spectrometer (HIDEX) with quantification from a linear calibration curve in the range of 20-800 Bq per sample (*r*^2^ > 0.999). The scintillation vials and the scintillation cocktail were purchased from Perkin Elmer.

An Ez-pi+ surface tensiometer, with dyneprobe (diameter: 0.51 mm) and polypropylene sample cuvettes were purchased from Kibron to measure the surface tension.

Liposomes were prepared using a mini-extruder (Avanti Polar Lipids) or a LIPEX Thermobarrel Pressure Extruder (10 mL, Northern Lipids). The hydrodynamic size and zeta potential of liposomes were measured by dynamic light scattering (DLS) on a Zetasizer (Malvern). Intensity weighted lognormal mean sizes are reported, unless otherwise noted. Sizes were measured in ISO-HEPES-NaCl and zeta potentials were measured in ISO-HEPES-Sucrose. Lipid concentrations were measured by ICP-MS (Thermo Scientifc, iCAP Q) by quantifying the phosphorous content of liposome samples against an internal standard of gallium (10 ppb). Sephadex G-25 PD-10 columns (8.3 mL bed volume) were purchased from GE Healthcare.

### Preparation of ^64^Cu

^64^Cu was produced by proton irradiation of an electroplated ^64^Ni target, and purified by anion exchange chromatography in aqueous HCl media. The ^64^Cu was ultimately obtained in aq. HCl (1.0 M), and dried under argon flow for use in radiolabeling, as previously described 10.

### Synthesis of D3R-C16 and D3R-C18

The tetrapeptide H-Arg(Pbf)-Arg(Pbf)-Arg(Pbf)-Lys(Mtt) was synthesized on an Initiator Alstra peptide synthesizer (Biotage, Uppsala, Sweden) using a novasyn TGR resin (loading 0.2 mmol/g). The resin was swelled in dichloromethane (DCM) for 1 hour. Each residue was coupled for 30 minutes at room temperature using 4 eq. amino acid, 3.92 eq. HATU and 8 eq. 2,4,6-collidine in *N*,*N*- dimethylformamide (DMF). Fmoc deprotection was done using 20% piperidine in DMF for 3 plus 10 minutes. Afterwards, either stearic acid or palmitic acid was coupled using 4 eq. of fatty acid, 3.92 eq. HATU and 8 eq. 2,4,6- collidine in DCM:DMF (1:1) for 60 min. Mtt-deprotection was obtained using 2% trifluoroacetic acid (TFA) in DCM. 25 washes of 10 mL for 5 min each were used. DOTA was coupled using 4 eq. of DOTA-tris-tBu, 3.92 eq. HATU and 8 eq. 2,4,6- collidine in DMF for 1 hour. The final products were cleaved for 3 hours using TFA/water/triisopropyl silane (95:2.5:2.5) after which the cleavage solvent was partially removed under reduced pressure and the peptide precipitated in diethyl ether. The cleaved products were dissolved in DMSO:water (5:95) and purified using semi-preparative HPLC (Waters 600 Pump & Controller and a Waters 2489 UV/Visible Detector). The C18 conjugate was purified employing a Waters XTerra^®^ C_18_ 5 µm (30 x 250 mm) column. Eluent: (A) 5% acetonitrile, 0.1% triethylamine (TFA) in water, (B) 0.1% TFA in acetonitrile. Gradient profile: Linear gradient from 25% B to 45% B over 40 min. Flow rate; 40 mL/min. The product was collected from 18-30 min. The C16 conjugate was purified employing a Waters XTerra^®^ C_18_ 5 µm (19 x 150 mm) column. Eluent: (A) 5% acetonitrile, 0.1% triethylamine (TFA) in water, (B) 0.1% TFA in acetonitrile. Gradient profile: Linear gradient from 20% B to 40% B over 20 min. Flow rate; 17 mL/min. The product was collected from 12-15 min. The products were lyophilized to obtain a white powder and the overall yield was 250 mg. The purity of the products was monitored by analytical HPLC using a Waters XBridge^®^ C_18_ 5 μm (4.6 x 150 mm) column. Eluent: (A) 5% acetonitrile, 0.1% TFA in water, (B) 0.1% TFA in acetonitrile. Gradient profile: Linear gradient from 0% B to 100% B over 15 min. Flow rate; 1 mL/min. Purity >95%. Rt. 11.0 min (C16) and 11.1 (C18). MALDI-TOF MS (Bruker Reflex, Bruker Daltonics, Billerica, MA, USA) (positive mode): Calc. M+H^+^: 1238.8 Da., Obs. M+H^+^: 1238.8 Da. (D3R-C16). Calc. M+H^+^: 1266.9 Da., Obs. M+H^+^: 1266.9 Da. (D3R-C18). For MALDI-TOF spectra see **Supplementary Information S2**.

### Cytotoxicity of D3R-C16 and D3R-C18

Cells were plated onto 96 well plates at a density of 4000 cells/well the day before the start of experiment. The original culture medium was replaced by medium containing increasing amounts of the indicated agents. After 24 h incubation with D3R-C16 or D3R-C18, the medium was removed and the cells were further incubated for another 48 hours. To measure metabolically active cells, 100 µL of MTS solution was added to the wells and incubated until sufficient amount of color was developed and within the linear range. The absorbance was measured at 490 nm using a microplate reader (Sunrise, Tecan).

### Surface tension measurements

D3R-C16 or D3R-C18 was dispersed in ISO-HEPES-NaCl or ISO-HEPES-Sucrose for a final concentration of 250 μM as stock solutions. Sample series were prepared by diluting the stock solutions with ISO-HEPES-NaCl or ISO-HEPES-Sucrose into concentrations of 125, 62.5, 31.25, 15.63, 7.81, 3.91, 1.95, 0.98 and 0.49 μM. The surface tension of the sample was measured by the Du Noüy method with a platinum probe. For each sample, the measurements were repeated until three consecutive measurements varied with less than 1 mN/m between them. Based on the critical micelle concentration of D3R-C18, a micellar dispersion of D3R-C18 in ISO-HEPES-Sucrose (2.0 mM) was prepared. The zeta potential of the D3R-C18 micelles was measured on a Zetasizer (Malvern).

### Radiolabeling of D3R-C16 or D3R-C18

A micellar dispersion of D3R-C16 or D3R-C18 in ISO-HEPES-NaCl (2.0 mL, 200 µM) was added to dried ^64^CuCl_2_ (about 300 MBq) or dried ^177^LuCl_3_ (about 140 MBq). The resulting mixtures were magnetically stirred at 55 ℃ (with ^64^CuCl_2_) or 90 ℃ (with ^177^LuCl_3_) for 30 minutes. The formation of ^64^Cu-D3R-C16 and ^64^Cu-D3R-C18 was confirmed by comparing the obtained retention factor (R*_f_* = 0.30 - 0.40, and R*_f_* = 0.40 - 0.50, respectively) with that of a non-radioactive chemically identical reference compound (for preparation, **see Supplementary Information S1**). The R*_f_* values for ^177^Lu-D3R-C16 were 0.2 - 0.25, and the R*_f_* values for ^177^Lu-D3R-C18 were 0.15 - 0.2. Free ^64^Cu and^ 177^Lu both remained at the origin (R*f* = 0). The radiochemical purity (RCP) was > 95% for all samples.

### Preparation of liposomes for membrane partitioning studies

Three different liposomes types were prepared: (1) POPC only, (2) POPC-POPG (9:1), (3) commercially available stealth lipid mixture (*see above*). POPC or the POPC-POPG mixture was freeze- dried from tert-butanol:water (9:1). The resulting lyophilizate or the stealth lipid mixture (187.5 mg) was then hydrated with ISO-HEPES-NaCl (5.0 mL) at 65 ℃ for 60 minutes by magnetic stirring. This was followed by sizing through a mini-extruder with a cut-off size of 100 nm. Size, PDI and zeta potential of all three preparations were measured (**Table 1**). The liposomes were diluted to lipid concentrations of 0.4, 1.0 and 4.0 mM for partitioning studies.

### Partitioning of D3R-C16 and D3R-C18 radiolabeled with ^64^Cu or ^177^Lu into liposomes

#### ^64^Cu-labeled

A dispersion of ^64^Cu-D3R-C16 (200 μM) in ISO-HEPES-NaCl (100 µL) was added to POPC liposomes (900 µL, 0.4 mM). The resulting mixture was magnetically stirred at 37 ℃. Mixtures were separated by size-exclusion chromatography (SEC) at 10, 30, 60, 120 and 180 minutes after addition. An aliquot (100 µL) was applied to a PD-10 cartridge and eluted with ISO-HEPES-NaCl. Two consecutive fractions of 5.5 and 4.5 mL were collected. The radioactivities of the two collected fractions and the column were measured by dose calibrator. The liposomal fraction eluted in the first 5.5 mL, whereas free ^64^Cu-D3R-C16 was retained on the column. The partitioning of the radiolabeled constructs into liposomes was calculated as the ratio of the radioactivity in the liposomal fraction to the total radioactivity of the liposomal fraction plus the column. Next, the partitioning of ^64^Cu-D3R-C16 and ^64^Cu-D3R-C18 into liposomes with different lipid compositions and different concentrations was studied. A dispersion of ^64^Cu radiolabeled D3R-C16 or D3R-C18 (200 mM, about 6 MBq) in ISO-HEPES- NaCl (30 µL) was added to POPC, POPC-POPG (9:1) or stealth liposomes (270 µL, 0.4 mM, 1.0 mM or 4.0 mM) respectively. The resulting mixtures were magnetically stirred at 37 ^o^C for 60 minutes. The mixtures were analyzed by SEC by applying 200 µL to a PD-10 cartridge (see above). The liposomal fraction eluted in the first 5.5 mL, whereas free ^64^Cu-D3R-C16 and ^64^Cu-D3R-C18 were retained on the column.

#### ^177^Lu-labeled

An ISO-HEPES-NaCl dispersion containing the ^177^Lu-D3R-C16 (30 µL, 200 μM) or ^177^Lu-D3R-C18 (30 µL, 200 μM) was added to stealth liposomes (270 µL, 0.4 mM, 1.0 mM or 4.0 mM). The resulting mixtures were magnetically stirred at 37 ℃ for 60 minutes. The mixtures were analyzed by SEC by applying 200 µL to a PD-10 cartridge as described above.

### Preparation of radioactive samples for *in vivo* experiments

The radioactivity concentration of all the ^64^Cu radiolabeled samples was 40 MBq/mL at the time of injection.

#### Free ^64^Cu

ISO-HEPES-NaCl (500 µL) was added to dried ^64^CuCl_2_ (160 MBq) and the mixture was stirred at 55 ℃, for 60 minutes. 50 µL of the sample was pipetted into a glass vial and the removed radioactivity was measured. The transfer efficiency, expressed as the ratio of the measured radioactivity to the theoretical maximum radioactivity of the withdrawn sample, was calculated to be 84%. The sample was then diluted with additional ISO-HEPES-NaCl (250 µL) to a concentration of 40 MBq/mL.

#### ^64^Cu-DOTA

A solution of DOTA (100 μM) in ISO-HEPES-NaCl (1.1 mL) was added to dried ^64^CuCl_2_ (160 MBq) and the mixture was stirred at 55 ^o^C for 60 minutes. 100 µL of the sample was withdrawn and the radioactivity was measured. The transfer efficiency was 96%. Radio-TLC was conducted and the R*_f_* of the ^64^Cu-DOTA complex was 0.3-0.4 with a radiochemical purity of >99%.

#### ^64^Cu-D3R-C18

A dispersion of D3R-C18 (100 μM) in ISO-HEPES-NaCl (1.1 mL) was added to dried ^64^CuCl_2_ (160 MBq) and the mixture was stirred at 55 ^o^C for 30 minutes. The transfer efficiency was 91%. Radio-TLC confirmed the formation of ^64^Cu-D3R-C18 in >95% RCP.

#### Liposomes loaded with ^64^Cu-D3R-C18 (LIP)

A dispersion of D3R-C18 (200 μM) in ISO-HEPES-NaCl (500 μL) was added to dry ^64^CuCl_2_ (160 MBq) and the mixture was stirred at 55 ℃ for 30 minutes. Radio-TLC confirmed the formation of ^64^Cu-D3R-C18 in >95% RCP. Empty stealth liposomes had been prepared by hydrating the purchased stealth lipid mixture with ISO-HEPES-NaCl and sizing using a LIPEX Thermobarrel Pressure Extruder, as described above. The mean diameter of the resulting liposomes was 115 nm (PDI = 0.033) and the zeta potential was 12.7±1.3 mV. The lipid concentration was 68 mM. ISO-HEPES-NaCl containing empty stealth liposomes (550 µL, 20 mM) was added to the ^64^Cu-D3R-C18 suspension (200 mM, 550 µL). The mixture was stirred at 55 ^o^C for 60 minutes. PD-10 analysis was conducted as described above and showed 98% partitioning of the radioactivity into the liposomes.

### Murine cancer model

All experimental procedures were approved and conducted under the guidelines of The Danish Animal Experiments Inspectorate. CT26 wildtype cells (murine colon carcinoma, ATCC, Virginia, US) were cultured RPMI (Roswell Park Memorial Institute) medium supplemented with 10 % heat-inactivated Fetal Calf Serum (FCS) and 1 % Penicillin-Streptomycin (Invitrogen, Carlsbad, CA., US) retained in 5 % CO_2_ incubator at 37 °C. Immunocompetent female Balb/C mice (Charles River, Wilmington, MA., USA) were inoculated with tumor cells after one week of adaptation. All 20 mice included were inoculated subcutaneously with 3 x 10^5^ CT26 wildtype cells in 100 µL medium on the right flank and hereafter monitored for weight and tumor size continuously for study purposes and to monitor the health of the mice. Tumor sizes were calculated from the formula of 0.52 x lenght^2^ x width, as measured by external caliper. Animals were included in the study when tumors were approximately 400 mm^3^. Four different ^64^Cu-compounds were investigated by PET/CT scanning to verify the diffusion after intratumoral injection: Free ^64^Cu (n=4), ^64^Cu-D3R-C18 (n=6), ^64^Cu-DOTA (n=4), and LIP (n=6).

### PET imaging and data analysis

Mice were injected intratumorally with ^64^Cu-compounds in concentrations of 40 MBq/mL. During the scan procedures, mice were anaesthetized using 3-4 % sevoflurane (Abbott Scandinavia AB, Sweden) mixed with 35% O_2_ and 65% medical grade air and placed on heating pads. Two mice were positioned side-by-side, separated by a 6 mm polystyrene block for PET/CT imaging using Inveon® small animal PET/CT system with CT based PET image attenuation (Siemens Medical Systems, Malvern, PA, USA). The protocol included a 12-minute PET scan, followed by a CT scan. Mice were fixed on a bed inside the scanner with the syringe placed and the needle fixed centrally in the tumor and ready for injection. Upon initiation of the first scan, intratumoral injections were done manually over 30 seconds, to investigate the initial dynamics of the tracer diffusion. Following this, two further scans were included; at six hours (5 minutes scan time) and 24 hours (12 minutes scan time) after injection. Reconstruction of the PET scans were performed using maximum a posteriori (MAP) reconstruction algorithm (voxel size: 0.815 x 0.815 x 0.796 mm; resolution (FWHM) 1.2 mm). The injection scan was reconstructed in 4 x 30 second and 10 x 60 second time frames to observe the dynamics of tracer diffusion. The two last time points were reconstructed in a single time frame.

Image analysis was performed using commercially available Inveon software (Siemens Medical Systems, Malvern, PA, USA). Regions of interest (ROIs) were manually drawn based on the co-registered PET/CT images. The following ROIs were constructed; tumors (complete volume delineated), liver, spleen, kidney, bladder, and blood. Blood activity was estimated from a constructed ROI covering the left ventricular lumen of the heart. ROIs in the left ventricle and abdominal aorta were subsequently segmented to only include the voxels displaying above 80% of maximum activity with the original ROI. From the obtained uptake values all data was calculated into %ID/g for each organ and as %ID in tumors by adjusting for CT based tumor volume. Furthermore, voxel values from tumor ROIs were used to generate histograms for substance diffusion over time in the tumor region.

### Well counting

After the completion of PET/CT imaging, mice were sacrificed and tumor, spleen, and liver tissue were collected and well counted (Wizard, Perkin Elmer, US). Tissue radioactivity was recorded and corrected for isotope decay and well counter efficiency. Final results are presented in %ID/organ calculated from activity and the tissue weight.

### Statistics

The statistics on *in vitro* data were calculated using Tukey's multiple comparison test with *post hoc* two-way ANOVA. For the *in vivo* data, the statistics were calculated by one-way ANOVA with *post hoc* Tukey's multiple comparison test when comparing the different preparations and by paired t-test for comparing intratumoral activity between the 2- and 12-minute time points for individual formulations. Non-paired multiple t-tests were used for comparing data for partitioning of surfactants into liposomes. Probability values below 0.05 (*p* < 0.05) were considered statistically significant and all analyses were performed in PRISM (version 7.04).

## Results

### Synthesis of non-radioactive Cu-D3R-C16 and Cu-D3R-C18 and their cytotoxicity

In order to confirm the identity of ^64^Cu-D3R-C16 and ^64^Cu-D3R-C18, the non-radioactive reference compounds Cu-D3R-C16 and Cu-D3R-C18 were prepared by methods analogous to the radiolabeling itself **(Supplementary Information, S1)**. Analysis by TLC gave R*f* values 0.3-0.4 for Cu-D3R-C16 and 0.4-0.5 for Cu-D3R-C18. The radiolabeling of both compounds gave 99% radiochemical purity (RCP) within 30 minutes. The radioactive products had the same R*f* values as the non-radioactive reference compounds, demonstrating the formation of ^64^Cu-D3R-C16 and ^64^Cu-D3R-C18. Free ^64^Cu stayed at the origin. To evaluate the stability and specificity of the radiolabeling, free DOTA or EDTA was added as challenge to both products in a molar ratio of 10:1 with DOTA or EDTA in excess. There was no detectable formation of ^64^Cu-DOTA (Rf = 0.3-0.4) or ^64^Cu-EDTA (Rf = 0.7-0.8) in both samples after 24 hours at room temperature, demonstrating that ^64^Cu was specifically chelated as the desired products.

As intratumorally administered D3R-C16 and D3R-C18 may migrate to surrounding healthy tissue, the toxicity of the compounds was investigated. The cytotoxicity of D3R-C16 and D3R-C18, as well as of non-radioactive Cu-D3R-C16 and Cu-D3R-C18, were measured towards CT26 cells **(Supplementary Information, S3)**. The median lethal concentration (LC_50_) of both D3R-C16 and D3R-C18 was found to be above 100 µM. In the animal study, a concentration of 100 µM D3R-C18 was injected intratumorally, as a dispersion of the free compound or formulated with liposomes (injection volumes: 50 µL). The tumor volumes were 430 ± 170 mm^3^ (n = 5) and 525 ± 82 mm^3^ (n = 4), respectively, based on CT. Accordingly, a lower bound estimate of the average concentration of D3R-C18 in the tumor is approximately 10 μM, assuming homogenous intratumoral distribution of the compound. Most likely the compound will distribute into the cellular membranes and lumen of the interstitial space, and the local concentration will be lower than 100 µM. This indicated that D3R-C18 is likely to have negligible local cytotoxicity towards the cancer cells. Furthermore, the probability of D3R-C18 accumulating at levels higher than the LC_50_ in healthy tissues is also considered unlikely.

### Physicochemical characterization of surfactants

**Figure 2:**
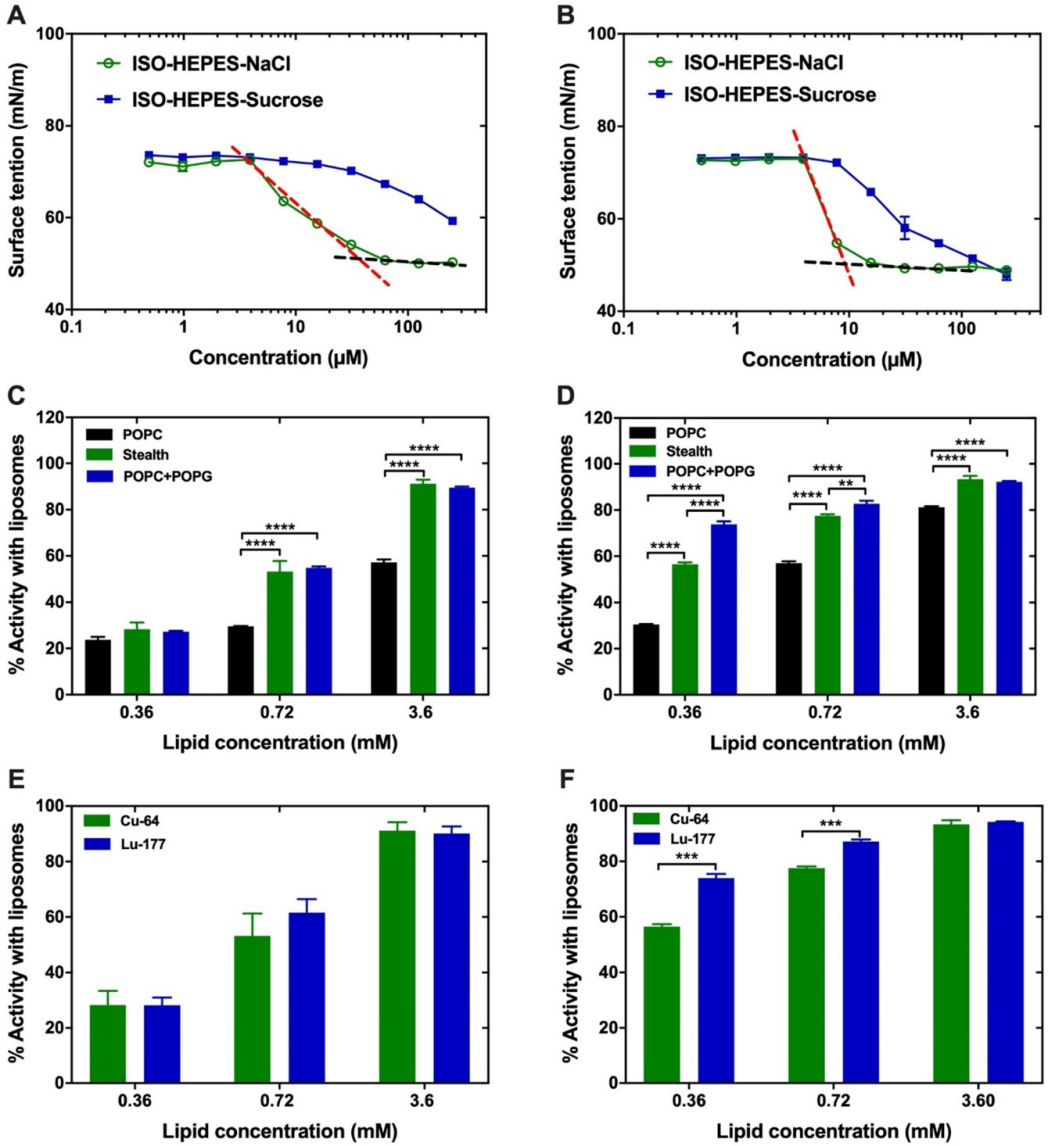
**Characterization of the D3R-alkyl compounds. (A,B)** Surface tension plotted against the concentration of (A) D3R-C16 and (B) D3R-C18. The surface tension is linearly dependent on the logarithm of the surfactant concentration in a range. Above CMC, the surface tension is independent of the surfactant concentration. The CMC is the intersection between the regression straight line (red dashed lines) of the linearly dependent region and the straight line passing through the plateau (black dashed lines). **(C,D)** Partitioning into liposomes of (C) ^64^Cu-D3R-C16 and (D)^ 64^Cu-D3R-C18. **(E,F)** Comparison of stealth liposome partitioning of (E) D3R-C16 and (F) D3R-C18 labeled with ^64^Cu^2+^ or ^177^Lu^3+^. Results are given as mean ± standard error of the mean (SEM). Significantly different values (p < 0.05) are shown as ** (p = 0.0026), *** (p < 0.0006) and **** (p < 0.0001).

D3R-C16 and D3R-C18 were designed as surfactants, due to their spatially separated hydrophobic alkyl chains and charged polar head groups. The sizes of the micelles formed by both compounds (2.0 mM) in ISO-HEPES were measured by DLS and showed broad distributions with an average size of 10 nm (Z-average) and PDI of 0.3, in both cases. In order to predict the behavior of D3R-C18 and D3R-C16 *in vivo*, the CMC and the partitioning into liposomes were investigated**.** The change in surface tension of the medium as a function of the concentration of D3R-C16 and D3R-C18 in ISO-HEPES-NaCl or ISO-HEPES-Sucrose was measured **(Figure 2A-B)**. The surface tensions decreased with the increasing accumulation of surfactant at the surface until they reached a plateau level, caused by formation of micelles. The CMC of both compounds was lower in ISO-HEPES-NaCl (D3R-C16: about 40 µM, D3R-C18: about 10 µM) than in ISO-HEPES-Sucrose, in which it was above 100 µM for both compounds. This can be attributed to electrolytes in the ISO-HEPES-NaCl screening the electrostatic charge repulsion between the ionized polar heads of the compound, causing micelles to form at lower surfactant concentrations. D3R-C18 exhibited a generally lower CMC than D3R-C16, likely due to the longer hydrophobic alkyl chain. The zeta potential of D3R-C18 micelles was 20.7 ± 0.5 mV.

D3R-C16 and D3R-C18 were radiolabeled with ^64^Cu for membrane partitioning studies. Liposomes were utilized as a prominent and widely used model for cell membranes 14. The kinetics of the partitioning were initially assessed in order to elucidate the incubation time needed for reaching partitioning equilibrium. The least hydrophobic surfactant, ^64^Cu-D3R-C16 was incubated at 37 ^o^C with neutral POPC liposomes at a low lipid concentration of 0.36 mM, which is considered the slowest of the partitioning studies due to the choice of membrane, lipid concentration and D3R acyl-chain length. The partitioning equilibrium was reached within 10 minutes **(Supplementary Information, S4)**, and consequently, the total incubation time in subsequent studies was set to 60 minutes to ensure equilibrium. The partitioning of both radiolabeled compounds into the lipid membrane increased with the liposome concentration regardless of liposome composition **(Figure 2C-D)**. At low lipid concentrations (0.36 mM), the lipid composition of the liposomes had no significant effect on the partitioning of ^64^Cu-D3R-C16 (POPC: 24 ± 1%, POPC+POPG: 27.2 ± 0.4%, Stealth: 28 ± 3%), which may be explained by partial charge neutralization of the anionic liposome. However, at lipid concentrations of 0.72 mM and 3.6 mM, the partitioning ratio of ^64^Cu-D3R-C16 into stealth liposomes (0.72 mM: 53 ± 5% and 3.6 mM: 91 ± 2%) and POPC+POPG liposomes (0.72 mM: 54.8 ± 0.7%, 3.6 mM: 89.5 ± 0.5%) was significantly higher than into POPC liposomes (0.36 mM: 29.6 ± 0.1%, 0.72 mM: 57 ± 1%). At lipid concentrations of 0.36 and 0.72 mM, the partitioning of ^64^Cu-D3R-C18 into POPC+POPG liposomes (0.36 mM: 74 ± 1%, 0.72 mM: 83 ± 1%) was higher than into stealth liposomes (0.36 mM: 56.5 ± 0.8%, 0.72 mM: 77.6 ± 0.6%), and even higher than into POPC liposomes (0.36 mM: 30.5 ± 0.3%, 0.72 mM: 57.0 ± 0.8%). However, no significant difference between ^64^Cu-D3R-C18 partitioning into POPC+POPG liposomes (92.1 ± 0.4%) or stealth liposomes (93 ± 1%) was observed at a lipid concentration of 3.6 mM. Furthermore, partitioning of ^64^Cu-D3R-C18 into POPC is significantly lower than the other two liposome types (81.2 ± 0.4%) at 3.6 mM lipid concentration. The cause of the lower partitioning of both radiolabeled D3R-alkyl compounds into POPC liposomes may be attributed to the neutral surface charge of these liposomes (-2.3 ± 0.6 mV). This results in lower affinity for the positively charged tri-arginine groups than in the case of the highly negatively charged surface of POPC+POPG liposomes (-49.1 ± 0.6 mV). In contrast, the partitioning of both ^64^Cu-D3R-C16 and ^64^Cu-D3R-C18 into stealth liposomes, that are also of limited zeta potential (-2.7 ± 0.3 mV), was high. This may be a consequence of negatively charged DSPE-PEG headgroups on the surface of these liposomes. The reason for the low zeta potential of stealth liposomes is masking of the charges by the PEG corona 15, 16. On this basis, the ^64^Cu-D3R-C16 and ^64^Cu-D3R-C18 appeared to have a significant affinity towards lipid membranes, with the tri-arginine group potentially enhancing the interaction with overall negatively charged lipid head groups on the surface. In comparison with ^64^Cu-D3R-C16, ^64^Cu-D3R-C18 showed higher partitioning into liposomes regardless of lipid composition and concentration, likely due to the enhanced hydrophobicity of the C18-moiety.

The partitioning of D3R-C16 and D3R-C18 labeled with two different radionuclides, ^177^Lu and ^64^Cu, was compared **(Figure 2E-F)**. There was no significant difference (*p* > 0.05) observed in the partitioning of D3R-C16 due to changing the radionuclides. However, the partitioning of ^177^Lu-D3R-C18 into lipid membranes was significantly higher than for ^64^Cu-D3R-C18 at low lipid concentrations (0.36 and 0.72 mM). The reason for this difference may be attributed to the trivalent ^177^Lu^3+^ forming a non-charged complex with DOTA, when one carboxylic acid on DOTA is used for conjugation. In contrast, divalent ^64^Cu^2+^ forms a negative complex with DOTA. Therefore, the ^177^Lu-DOTA moiety may have a higher affinity for negatively charged liposomal surfaces as compared to the ^64^Cu-DOTA moiety. These results suggest that the valence of the radionuclide in use should be taken into consideration when predicting the intratumoral behavior of the DOTA-alkyl compounds.

### *In vivo* evaluation of radiolabeled samples

^64^Cu-D3R-C18 was selected as the preferred candidate for establishing *in vivo* proof of concept, as the D3R-C18 analogue displayed the highest membrane partitioning, and ^64^Cu allows for quantitative biodistribution analysis based on PET/CT imaging. The high partitioning into liposomes indicated a potentially higher affinity towards cancer cell membranes, which could result in the desired longer intratumoral retention of the compound. ^64^Cu-D3R-C18 was administered intratumorally both as the free compound in the form of micelles and formulated with liposomes. Free ^64^CuCl_2_ and free ^64^Cu-DOTA were intratumorally administered as controls. According to the CMC of D3R-C18 of about 10 μM in ISO-HEPES-NaCl, the injected ^64^Cu-D3R-C18 (100 μM) was primarily composed of cationic micelles. Stealth liposomes with ^64^Cu-D3R-C18 (^64^Cu-LIP) were also evaluated *in vivo*. The post-insertion of ^64^Cu-D3R-C18 into the stealth liposomes occurred within 60 minutes with a quantitative loading efficiency of >98% at a lipid concentration of 10 mM.

**Figure 3:**
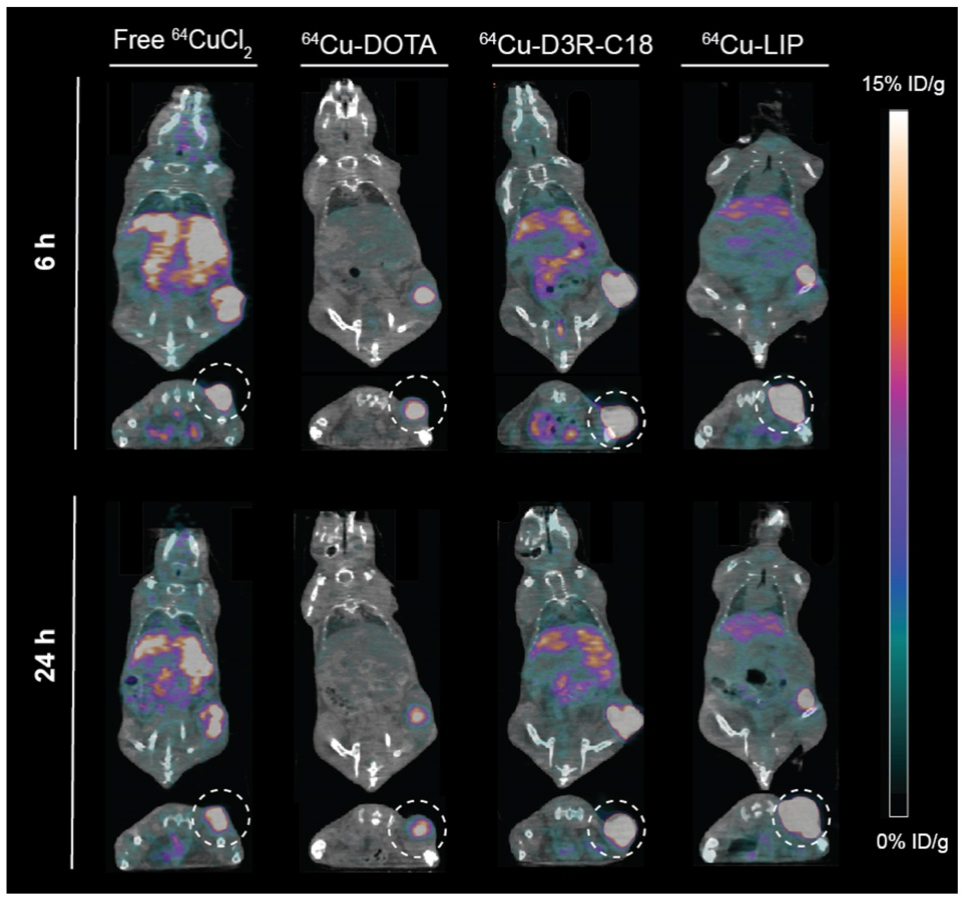
** Coronal and transverse PET/CT images of tumor bearing mice injected with free ^64^CuCl_2_, ^64^Cu-DOTA, ^64^Cu-D3R-C18 or ^64^Cu-LIP at 6 hours and 24 hours.** Tumors are highlighted in the transverse images by a dashed circle.

**Figure 4:**
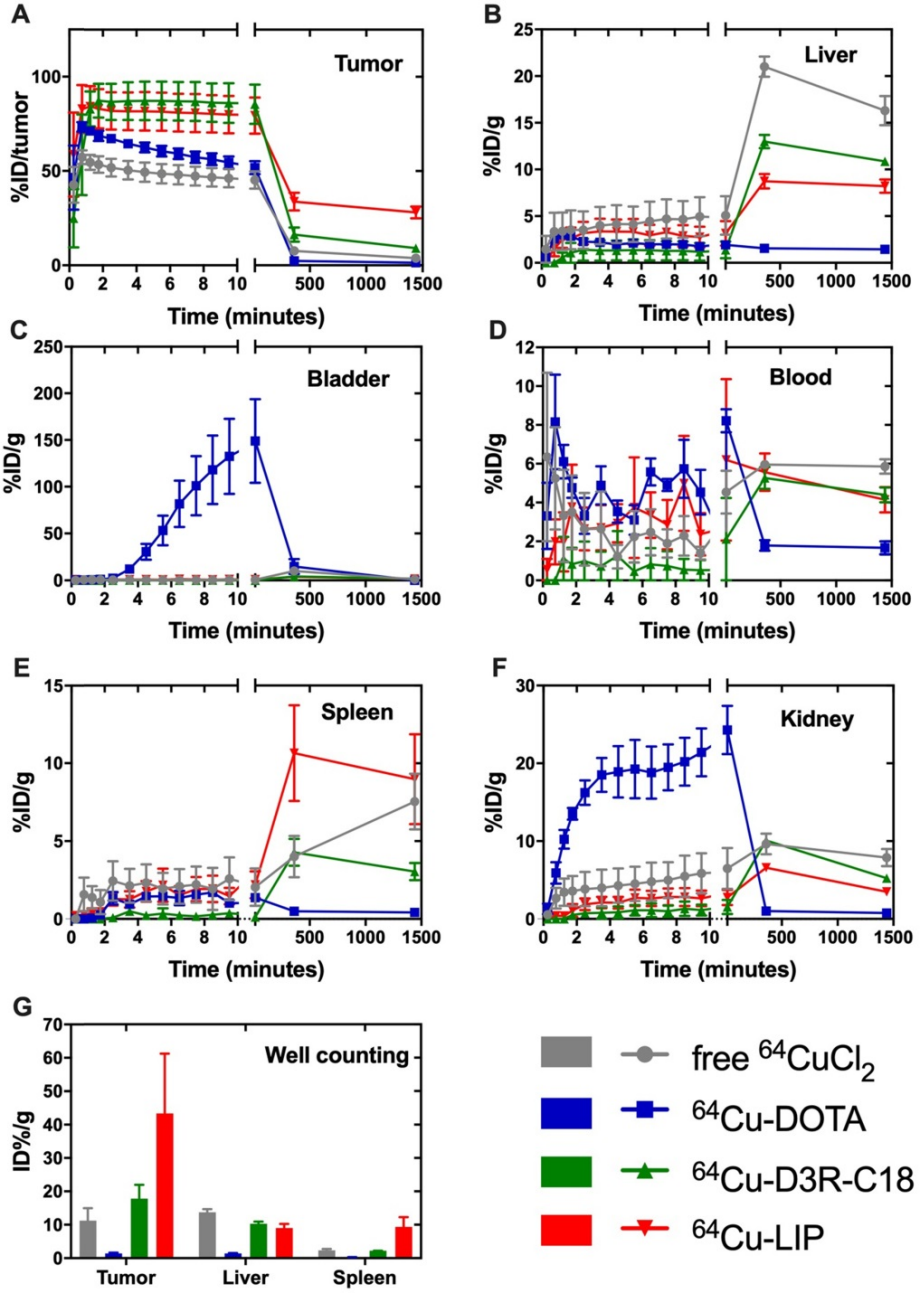
**Biodistribution of free^ 64^CuCl_2_, ^64^Cu-DOTA, free ^64^Cu-D3R-C18 and ^64^Cu-LIP in (A) tumor, (B) liver, (C) bladder, (D) blood, (E) spleen and (F) kidney based on PET imaging.** The results are reported as mean and SEM (Free ^64^CuCl_2_ and ^64^Cu-DOTA n = 3, ^64^Cu-D3R-C18 n = 5, and ^64^Cu-LIP n = 4). (F) *Ex vivo* biodistribution of free^ 64^CuCl_2_, ^64^Cu-DOTA, free ^64^Cu-D3R-C18 and ^64^Cu-LIP. The animals were sacrificed after the 24-hour scan and excised organs were well-counted. The results are reported as mean ± SEM (free ^64^CuCl_2_ and ^64^Cu-DOTA n = 3, ^64^Cu-D3R-C18 n = 5, and ^64^Cu-LIP n = 4).

The *in vivo* biodistribution of the four radiolabeled preparations was investigated by PET/CT imaging **(Figure 3)** and quantified as percent injected dose per gram tissue (%ID/g) for organs and as percent of total injected dose in tumors (%ID/tumor) **(Figure 4A-F)**. *Ex vivo* biodistributions were also determined by organ well counting **(Figure 4G)**. Figure 4A shows the tumor retention of the four preparations. Note that the initially increasing dose in the tumors is due to slow manual intratumoral injection, lasting about 30 seconds. ^64^Cu-DOTA was cleared very rapidly after injection from both the tumor and the body through the urinary system **(Figure 4C+F)**, in line with previous observations 17, 18. The intratumoral ^64^Cu-DOTA activity was already significantly lower at the 12 minute time point compared to the activity at 2 minutes (*p* = 0.014). At 12 minutes post injection, 52 ± 4 %ID/tumor ^64^Cu-DOTA was present in the tumor. Only 2.4 ± 0.7 %ID/tumor and 1.4 ± 0.4 %ID/tumor was found in the tumor at 6 hours and 24 hours respectively, underscoring the rapid wash-out. Free ^64^CuCl_2_ is known to accumulate in solid tumors after systemic administration 19, 20. Here however, we observed an initial tumor clearance with significantly lower activity at the 12 minute time point compared to 2 minutes after injection (*p* = 0.004), and the retention of free ^64^Cu was only 45 ± 5% ID/tumor at 12 minutes. The ^64^Cu was continuously cleared with tumor retention of 7.7 ± 0.6 %ID/g and 3.8 ± 0.5 %ID/g at 6 hours and 24 hours, respectively. In agreement with previous reports, most of the released free ^64^CuCl_2_ accumulated in liver and kidney 19. The retention of ^64^Cu-D3R-C18 micelles was not significantly reduced between the 2- and 12-minute time point, and the 12-minute time point displayed the highest activity of the four preparations (85 ± 11 %ID/tumor), although not significantly different from ^64^Cu-LIP. Unlike ^64^Cu-DOTA that had no significant accumulation in healthy tissues, ^64^Cu-D3R-C18 administrated as micelles accumulated gradually in liver, blood, spleen and kidney. The tumor retention of ^64^Cu-D3R-C18 might be due to its positive charge and acyl chain increasing the affinity towards the negative membrane of cancer cells. The initial tumor retention of ^64^Cu-LIP was comparable to ^64^Cu-D3R-C18 at 12 minutes (80 ± 10 %ID/tumor) and, as with the ^64^Cu-D3R-C18 formulation, no significant reduction in activity was observed between the 2- and 12-minute time points. The intratumoral retention of ^64^Cu-LIP and ^64^Cu-D3R-C18 was significantly higher than that of ^64^CuCl_2_ at the 12 minute PET scan (*p* = 0.046 and 0.019, respectively). As free ^64^Cu-D3R-C18 would be released from liposomes after the injection of the liposomal preparation, the clearance of radioactivity could be both in the form of free ^64^Cu-D3R-C18 and ^64^Cu-LIP. An initial clearance of liposomes from the tumor was also reported in previous studies 13, 21. After the initial clearance, the tumor retention of ^64^Cu-LIP stabilized at a level of 34 ± 5 %ID/tumor at 6 hours and 28 ± 3 %ID/tumor at 24 hours, which at both time points was significantly higher than the ^64^Cu-D3R-C18 (*p* = 0.012 and 0.0001), ^64^CuCl_2_ (*p* = 0.0006 and < 0.0001) and ^64^Cu-DOTA (*p* = 0.002 and < 0.0001) tracers. The cleared dose was primarily observed to accumulate in spleen and liver with a gradually increasing accumulation up to >5 %ID/g from 6 hours onwards, which is consistent with previous studies of ^99m^Tc-labeled liposomes by intratumoral injection 21. The long tumor retention time of ^64^Cu-LIP might be attributed to a combination of (1) slow clearance from the interstitial space because of the large liposome particle size 13, and (2) slow release of ^64^Cu-D3R-C18 from the liposomes when they were diluted after injection. The *ex vivo* data for the organ accumulation in each group **(Figure 4G)** showed a similar trend as the PET imaging data at 24 hours **(Figure 4A-F)**. The retained radioactivity in tumors given as %ID/g for each group is presented in **Supplementary Information, S5** and the weights of all tumors and organs, as well as %ID/g data from well counting, are listed in **Supplementary Information S6**.

### Intratumoral distribution of radiolabeled samples

**Figure 5:**
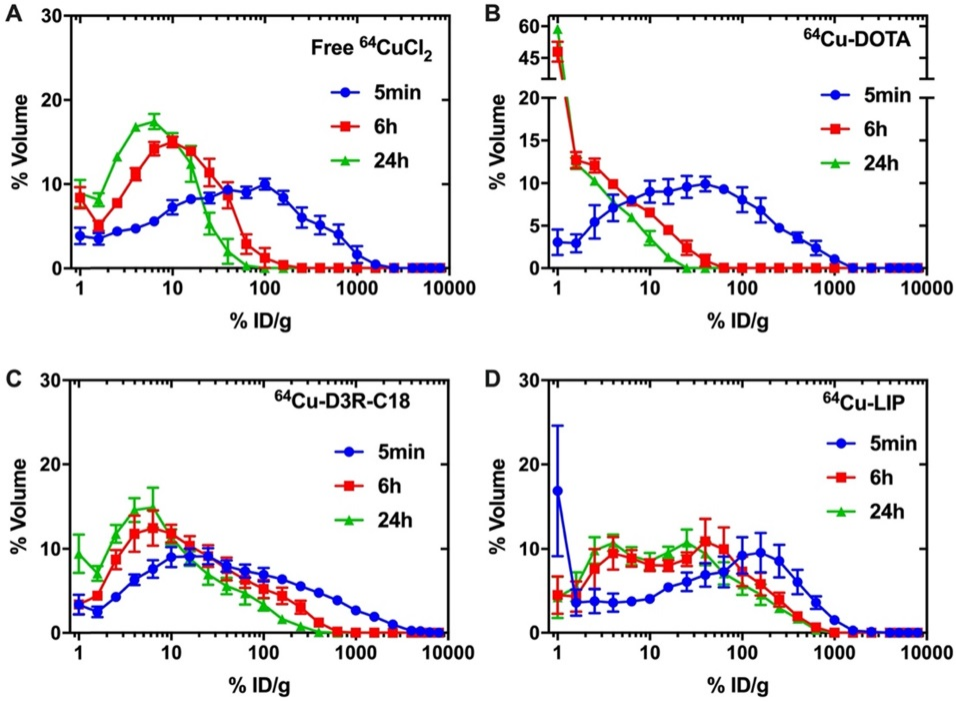
**Frequency distribution functions of %volume as a function of %ID/g for free ^64^CuCl_2_ (A), ^64^Cu-DOTA (B), ^64^Cu-D3R-C18 (C) and LIP (D).** Points on the curve present the mean-values of bin width. The first point for each frequency distribution (for each formulation) shows the percent voxels with % injected activity/mL lower than 1.6%. The results are reported as mean and SEM (^64^Cu free and ^64^Cu-DOTA: n = 3, ^64^Cu-D3R-C18: n=5, ^64^Cu-LIP: n = 4).

The distribution of radioactivity within the tumor was analyzed by plotting the radioactivity of each voxel as a frequency distribution **(Figure 5)**. Note that the first point in each plot represents the percentage of voxels with radioactivity concentrations lower than 1.6 %ID/g. These voxels are considered empty, meaning that the corresponding tumor region is non-radioactive. Free ^64^CuCl_2_, ^64^Cu-DOTA and ^64^Cu-D3R-C18 distributed more rapidly through the tumor with less than 10% non-radioactive voxels at 5 minutes after the start of the PET scan, as compared with more than 15% non-radioactive voxels in the ^64^Cu-LIP. More than 40% non-radioactive voxels were observed in the ^64^Cu-DOTA group at 6 h, illustrating the fast washout from the tumor. This corresponded with the relatively low tumor retention of this compound, as observed by PET **(Figure 3)**. A shift towards lower activity (left shift) of the frequency distributions were observed for all formulations from 5 min to 6 h, indicating that the remaining radioactivity distributed gradually throughout the tumor. Free ^64^CuCl_2_ exhibited a narrow radioactivity distribution with general voxel radioactivities from 0 to 100 %ID/g at 6 h, becoming even more constricted from 0 to 50% at 24 h. This demonstrated a fast distribution and wash-out from the tumor of free ^64^CuCl_2_. ^64^Cu-D3R-C18 distributed slower than free ^64^Cu, although still with a gradually left-shifting curve. However, a very wide range of distribution from 0 to about 500% ID/g at 24 hours was observed. The slowest intratumoral distribution occurred for the ^64^Cu-LIP formulation. Virtually no change in the distribution of ^64^Cu-LIP was observed between 6 h and 24 h, both time points with a wide distribution from 0%ID/g to about 800%ID/g. ^64^Cu-LIP displayed a distribution with double peaks at 6 h and 24 h, and the lower dose peak was similar to the peak of ^64^Cu-D3R-C18 at the same time point.

In summary, free ^64^CuCl, ^64^Cu-DOTA and ^64^Cu-D3R-C18 distributed very fast in 24 h. However, the fast distribution may be caused by rapid wash-out from the tumor. Therefore, combining the results of intratumoral retention and distribution, ^64^Cu-D3R-C18 appears to have the advantages of providing both good retention as well as distribution of radiation in the first 6 h, compared to the other test groups. ^64^Cu-LIP had the slowest intratumoral distribution among all the formulations. This may be due to the slow release of ^64^Cu-D3R-C18 from liposomes and the slow diffusion rate of liposomes in the tumors due to their larger size compared to small molecules.

## Discussion

Current BT technologies are highly invasive, requiring numerous implantations of solid radioactive sources. Novel liquid BT technologies that can be injected intratumorally at a single or a few sites, from where they would spread homogenously through the tumor to irradiate the entire lesion, are thus highly attractive. Such a system would also allow the use of alpha and beta emitting therapeutic radionuclides, many of which are currently becoming available in pharmaceutically relevant qualities. Complete tumor coverage is crucial in current brachytherapy systems and dosimetry is often challenging. The PET imaging properties of the presented system provide accurate information on intra- and extratumoral distribution, and allows for precise calculation of brachytherapy dosimetry. This can furthermore be incorporated in EBRT planning for optimal combination of the two modalities.

Liquid brachytherapeutic systems such as the D3R-C18 formulation can be injected using small gauge needles and should optimally distribute throughout the intended tumor region within a few hours. The radionuclides should then be well-retained with minimal wash-out. This would enable homogenous irradiation of the tumor tissue, and would permit the use of short-lived therapeutic radionuclides (T_½_ = 5-10 hours). These would irradiate the tumor for a prolonged period, but would have decayed significantly once inevitable escape from the tumor occurred. The flexibility of a chelator based delivery system further allows for the use of theranostic radionuclide pairs 22 enabling simple dosimetric mapping via PET or SPECT imaging.

In this study, we prepared two novel DOTA-based chelating agents containing hydrophobic acyl chains and tri-arginine groups. These were designed to be able to transition from cell membrane to cell membrane, carrying radioactive material from the injection point through tissue in a gradual and controlled fashion. Cancer cell surfaces are known to be more negatively charged than normal cells 23. Hence, highly positively charged tri-arginine groups were incorporated in order to increase the affinity to the negative lipid surface. The conducted partitioning studies of ^64^Cu-D3R-C18 into liposomes supported this hypothesis by showing higher affinity to membranes with a higher degree of negatively charged head groups.

By radiolabeling with ^64^Cu and imaging by PET/CT, the biodistribution of ^64^Cu-D3R-C18 was investigated. The compound was administered in the free form (^64^Cu-D3R-C18) and as a formulation in liposomes (^64^Cu-LIP). CMC measurements indicated that the free compound was present in the injection medium primarily as micelles. The radiolabeled compound was observed to exhibit substantial tumor retention, both as the free compound and in liposomes. In contrast, the control compounds, free ^64^CuCl_2_ and ^64^Cu-DOTA, exhibited rapid washout from the tumor. Although retention was observed for ^64^Cu-D3R-C18 during the initial 12 minute dynamic scan, there was a significant wash-out at 6 hours and 24 hours. On the contrary, the ^64^Cu-LIP displayed both limited initial clearance and more stable intratumoral levels of radioactivity at 6 and 24 hours. It is possible that this is due to slower clearance, due to the larger particle size 13. Both formulations showed some off-target accumulation in liver, spleen and kidneys, which is in accordance with well-established biodistribution profiles for nanoparticulate substance 24, 25. In this regard, it is important to note that the ^64^Cu-D3R-C18 micelles would start to disintegrate into unimers after injection, because of dilution below the CMC value. Single unimers entering the blood stream would be likely to exist as free molecules but may also be associated with albumin, which makes up about 60% of the total protein in the blood. The accumulation in liver, spleen and kidneys may be a result of association to this protein 26.

While tumor retention of injectable brachytherapy formulations has been addressed in other studies, the intratumoral distribution is seldom discussed. Previously, only rough estimations or comparisons of distribution between different formulations from PET images have been performed 13, 27. In our study, voxel activity histograms were constructed to quantify and provide detailed information on the intratumoral radioactivity distribution of each formulation. The radioactivity from both formulations was observed to gradually distribute through the tumor. According to the histograms, the distribution of ^64^Cu-D3R-C18 in micellar form is more homogenous than when formulated in liposomes (^64^Cu-LIP) in 24 hours. The heterogeneous distribution of radioactivity is caused not only by the formulation design, but also the histopathological heterogeneity of the tumor. Tumor heterogeneity includes differences in cells densities, microenvironmental factors such as hypoxia and interstitial fluid pressure, and vasculature 28. These features demonstrate the importance of PET tracking properties for determination of tumor coverage.

The nanoparticulate formulations investigated in this study, showed relatively high retention up to 6 hours after injection. Because of this, these agents are potentially best suited for intratumoral delivery of relatively short-lived therapeutic radionuclides, which is likely to exclude their use in combination with ^177^Lu for therapeutic purposes. Faster decaying radionuclides would prevent washout and off-target accumulation of high amounts of radioactive material. The radioactivity of both formulations was homogenously distributed in the injected tumors. This entails that short-ranged emissions may be applicable for these systems, making especially alpha-emitters relevant. Alpha particles are particularly cytotoxic because of the high linear energy transfer radiation 29. With half-lives of about 1 hour, the alpha-emitters ^212^Bi (T½ = 61 min) and ^213^Bi (T½ = 46 min) become relevant. The free D3R-C18 could be radiolabeled within < 1h and injected intratumorally as the free compound. On the other hand, liposomes loaded with D3R-C18 might be promising with longer-lived radionuclides, due to their longer retention in tumors of up to 24 hours. However, this would still incur significant irradiation of healthy tissue, as the liposomes are seen to accumulate in organs beyond the tumor after 6 hours. In humans, however, it is possible that this window could be extended, due to slower pharmacokinetics. Even though the intratumoral distribution of the liposome formulation was slower than D3R-C18, the relatively long range and crossfire effect of beta particles can still provide a fairly homogenous radiation dose 27. Since D3R-C18 showed a high initial accumulation in the tumor in the first 12 min (Figure 4A) and it inserts into membranes, using Auger therapy may also be potentially useful, since short-range electrons have been demonstrated to have a pronounced cytotoxic advantage from decaying in the membrane 30.

## Conclusion

We synthesized two novel non-toxic DOTA- triarginine-lipid conjugates, D3R-C16 and D3R-C18, to deliver radionuclides for brachytherapy. The delivery system used small gauge needles for injection and the compounds were designed to diffuse from cell membrane to cell membrane, to distribute radioactive material throughout the tumor. The *in vitro* CMC and partitioning into liposomes with different surface properties of D3R-C16 and D3R-C18 was investigated. The lead compound, D3R-C18, was radiolabeled with ^64^Cu (>99% RCP in 30 min) and evaluated *in vivo* by intratumoral administration as both free micelles and a formulation in liposomes and imaged by PET/CT. The micelle and liposome formulations both showed a high initial retention, and liposomes displayed a higher long-term retention but a slower intratumoral distribution than ^64^Cu-D3R-C18 in micellar form. Our results demonstrate the brachytherapeutic potential of liposomal and micellar formulations of D3R-C18, especially when paired with short-lived radionuclides.

## Supplementary Material

Supplementary figures and tables.

## Figures and Tables

**Table 1:** Properties of liposomes for partitioning studies

	POPC	POPC+10% POPG	Stealth
**Size (nm)**	120.6 ± 0.3	129.6 ± 0.9	134.2 ± 0.8
**PDI**	0.040 ± 0.02	0.07 ± 0.01	0.05 ± 0.02
**Zeta potential (mV)**	-2.3 ± 0.6	-49.1 ± 0.6	-2.7 ± 0.3

## Abbreviations

BT: brachytherapy
%ID/g: percent injected dose per gram
ANOVA: analysis of variance
CHOL: cholesterol
CMC: critical micelle concentration
CT: computed tomography
DLS: dynamic light scattering
DOTA: 1,4,7,10-tetraazacyclododecane-1,4,7,10-tetraacetic acid
DCM: dichloromethane
DMF: dimethylformamide
DSPE-PEG2000: 1,2-distearoyl-sn-glycero-3-phospho-ethanolamine-N-[methoxy(polyethyleneglycol)-2000]
DMSO: dimethyl sulfoxide
EBRT: external beam radiotherapy
EDTA: ethylenediaminetetraacetic acid
Fmoc: 9-fluorenylmethoxycarbonyl
HATU: *O*-(7-azabenzotriazol-1-yl)-1,1,3,3-tetramethyluronium hexafluorophosphate
HDR: high dose-rate
HEPES: 4-(2-hydroxyethyl)piperazine-1-ethanesulfonic acid
HPLC: high-performance liquid chromatography
HSPC: hydrogenated soy phosphatidylcholine
ICP-MS: inductively coupled plasma mass spectrometry
LBT: liquid brachytherapy
LC_50_: median lethal concentration
LDR: low dose-rate
RCP: radiochemical purity
PDI: polydispersity index
PEG: polyethylene glycol
PET: positron emission tomography
POPC: 1-palmitoyl-2-oleoyl-sn-glycero-3-phosphocholine
POPG: 1-palmitoyl-2-oleoyl-sn-glycero-3-phospho-(1-rac-glycerol)
R*_f_*: retention factor
ROI: region-of-interest
SEC: size-exclusion chromatography
SEM: standard error of the mean
T½: radioactive decay half-life
TFA: trifluoroacetic acid
TLC: thin-layer chromatography
