# Supplementary material for: Preclinical evaluation of cationic DOTA-triarginine-lipid conjugates for theranostic liquid brachytherapy
Source: Nanotheranostics. 2020 Apr 22;4(3):142–55. doi: 10.7150/ntno.44562 (PMC7256013; doi:10.7150/ntno.44562)
Supplement: Supplementary file 1 — Supplementary figures and tables. [file ntnov04p0142s1.pdf]

Supplementary information for

Preclinical evaluation of cationic DOTA-triarginine-lipid conjugates for theranostic liquid brachytherapy

Wenbo Wang[1,4], Frederikke P. Fliedner[2], Anders E. Hansen[1,2,4], Rasmus Eliasen[1,4], Fredrik Melander[1,4], Andreas Kjaer[2], Thomas L. Andresen[1,4], Andreas I. Jensen[3,4]*, Jonas R. Henriksen[1,4]*

[1] Department of Health Technology, Technical University of Denmark, Produktionstorvet Building 423, DK 2800 Lyngby, Denmark

[2] Rigshospitalet and University of Copenhagen, Dept. of Clinical Physiology, Nuclear Medicine & PET, Cluster for Molecular Imaging, 2100 Copenhagen, Denmark

[3] Center for Nuclear Technologies (DTU Nutech), Technical University of Denmark, Frederiksborgvej 399, DK, 4000 Roskilde, Denmark

[4]Center for Nanomedicine and Theranostics.

S1 – Preparation and TLC characterization of non-radioactive Cu-D3R-C16 and Cu-D3R-C18 reference compounds

Method

A micellar dispersion of D3R-C16 or D3R-C18 (200 μM) in ISO-HEPES (NaCl, 500 μL) was added to $CuCl_2$ (molar ratio Cu^{2+}:D3R-C16 or D3R-C18 10:1). The resulting mixtures were magnetically stirred at 55 °C for 30 minutes. 1 μL of D3R-C16, D3R-C18, Cu-D3R-C16 or Cu-D3R-C18 was spotted on silica gel 60 F254 plates (Merck) and a solution of 5% (w/v) ammonium acetate (NH_4OAC) in water-methanol (1:3) was used as eluent. All the compounds were observed by using permanganate stain.

Results

Analysis by TLC gave R*f* values 0.3-0.4 for Cu-D3R-C16 and 0.4-0.5 for Cu-D3R-C18. D3R-C16 and D3R-C18 have similar R*f* of 0.6-0.7.

S2 – MALDI-TOF spectra for D3R-C16 and D3R-C18

D3R-C16

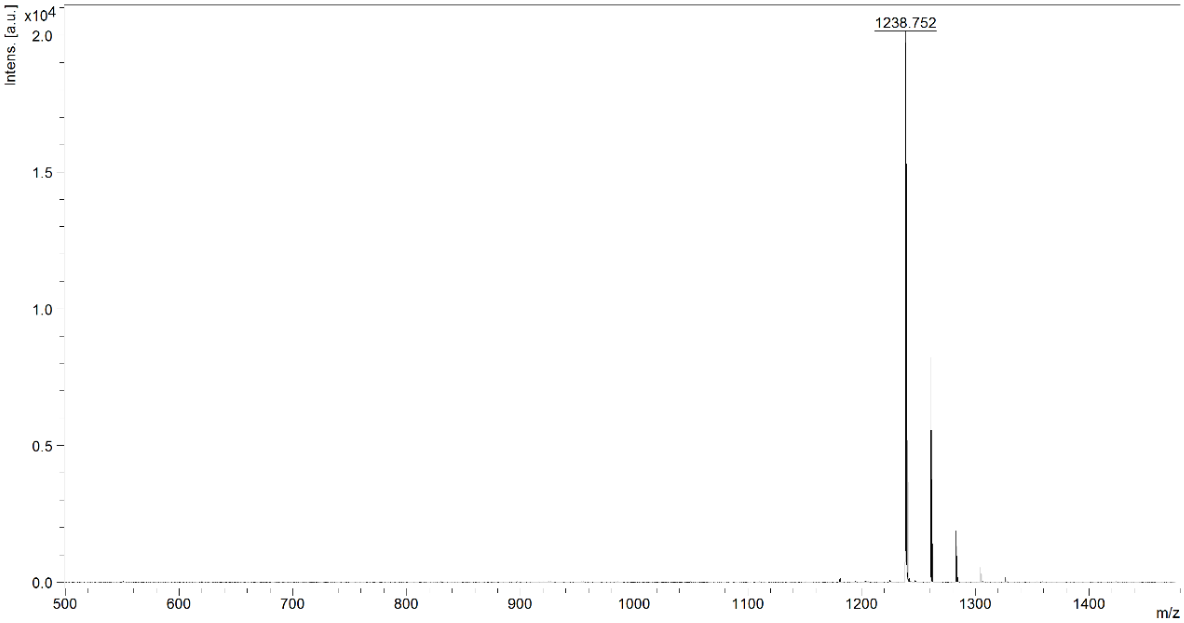

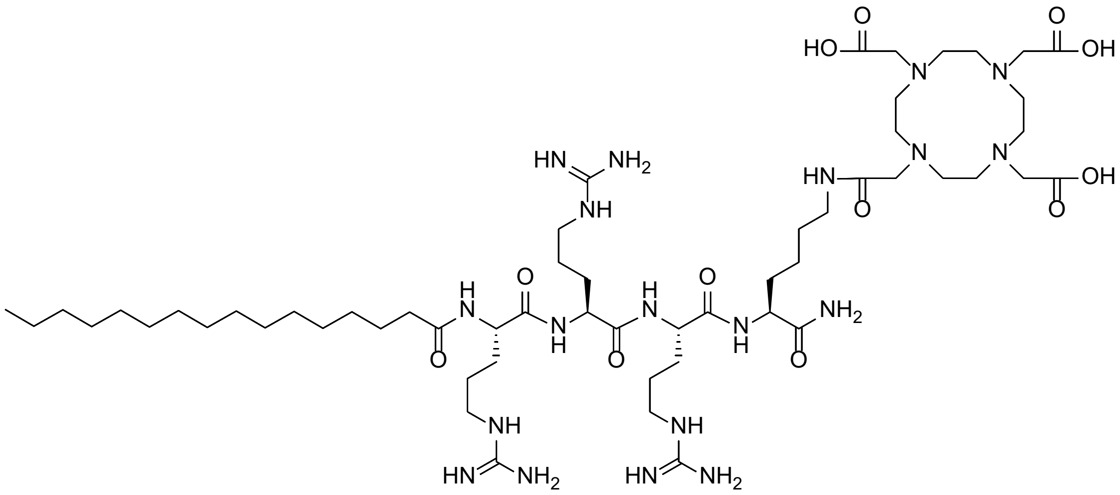

Exact Mass: 1237,83
Molecular Weight: 1238,59

D3R-C18

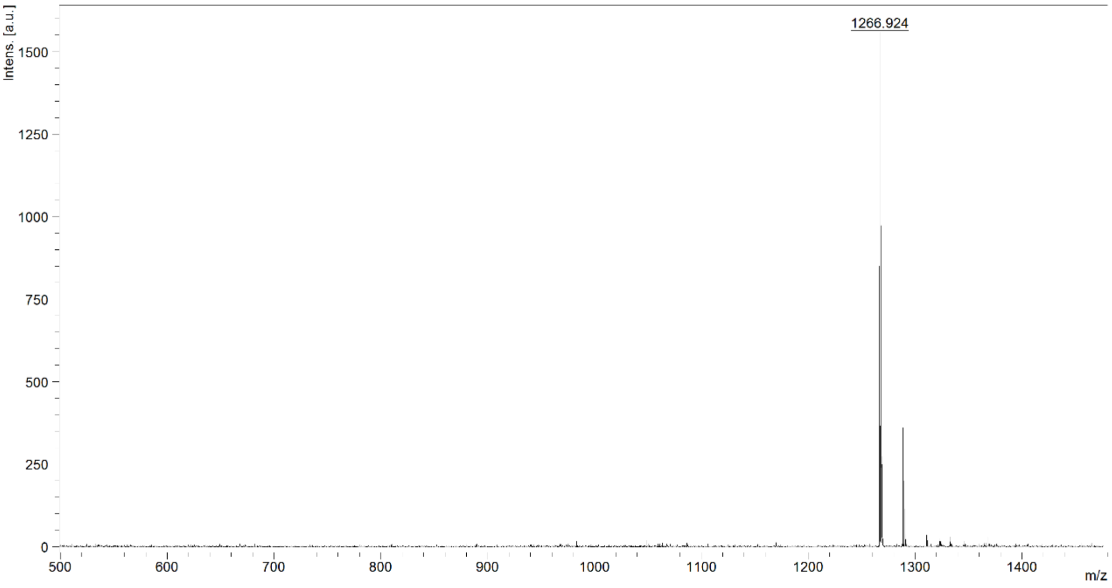

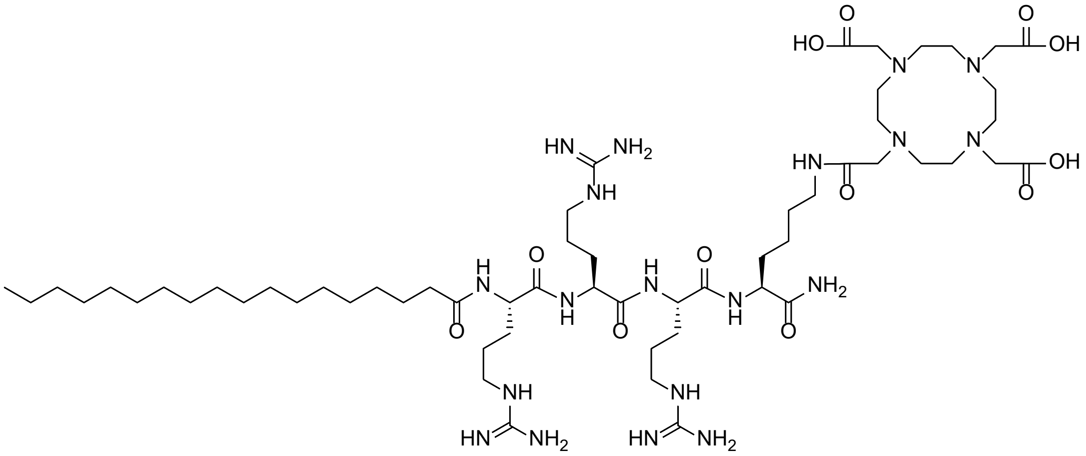

Exact Mass: 1265,87
Molecular Weight: 1266,65

S3 – Cytotoxicity of D3R-alkyl and non-radioactive Cu-D3R-alkyl

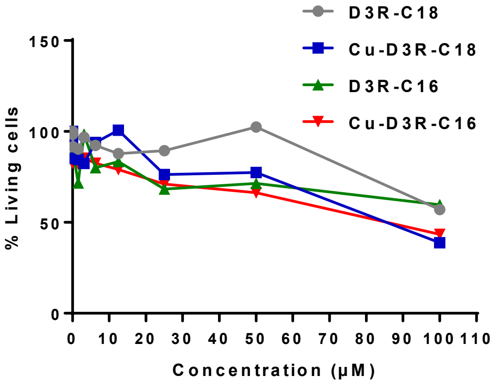

Figure S1. Cytotoxicity of C16/C18-3R-DOTA and their Cu^{2+} chelates with towards CT26 cells.

S4 – Partitioning kinetics of ^{64}Cu-D3R-C16 into POPC liposomes (0.36 mM)

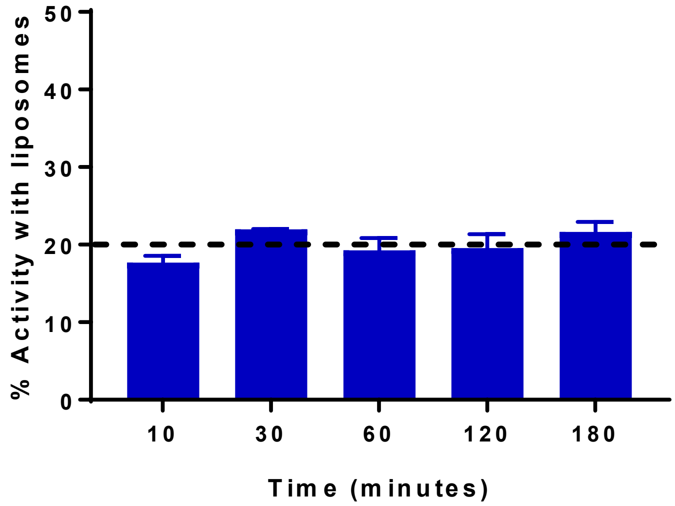

Figure S2. Partitioning kinetics of ^{64}Cu-D3R-C16 into liposomes as a function of time. The results are given as mean ± SEM (n = 3).

S5 – Absolute retention of radioactivity in tumors

Absolute retention of radioactivity (%ID/g) in tumors as a function of time. Data are presented as mean ± SEM.

Time	Free ^{64}Cu n = 3	^{64}Cu-DOTA n = 3	^{64}Cu-DOTA-3R-C18 n = 5	^{64}Cu-LIP n = 4
0.25 min	97 ± 19	78 ± 37	80 ± 66	104 ± 62
0.75 min	167 ± 50	130 ± 13	171 ± 96	157 ± 26
1.25 min	164 ± 53	127 ± 14	230 ± 58	161 ± 30
1.75 min	162 ± 54	123 ± 14	241 ± 63	159 ± 30
2.5 min	159 ± 53	120 ± 14	241 ± 66	157 ±30
3.5 min	156 ± 54	115 ± 14	240 ± 68	157 ± 30
4.5 min	154 ± 53	111 ± 14	241 ± 69	157 ± 31
5.5 min	152 ± 53	108 ± 14	240 ± 70	157 ± 31
6.5 min	150 ± 53	105 ± 14	240 ± 70	156 ±31
7.5 min	148 ± 52	102 ±14	240 ± 70	155 ± 31
8.5 min	146 ± 52	100 ± 14	239 ± 69	154 ± 31
9.5 min	145 ± 53	97 ± 13	238 ± 70	154 ± 31
10.5 min	143 ± 52	94 ± 13	238 ± 70	153 ± 31
11.5 min	142 ± 51	92 ± 12	236 ± 70	153 ± 30
6 h	18 ± 3	4 ± 1	49 ± 17	70 ± 22
24 h	10 ± 1	2 ± 0.8	23 ± 7	55 ± 13

S6 - Well counting results: weights of tumor and organs, and accumulation of radioactivity (%ID/g) in relevant organs

Weights (g) of tumor and organs. Data are presented as mean ± SEM.

	Tumor	Liver	Spleen
Free ^{64}Cu, n=3	0.14 ± 0.079	0.39 ± 0.091	0.15 ± 0.11
^{64}Cu-DOTA, n = 3	0.42 ± 0.069	0.43 ± 0.10	0.085 ± 0.0047
^{64}Cu-DOTA-3R-C18, n = 5	0.22 ± 0.094	0.37 ± 0.12	0.094 ± 0.0064
LIP, n = 4	0.34 ± 0.11	0.40 ± 0.12	0.090 ± 0.015

Accumulation of radioactivity (ID%/g) in relevant organs. Data are presented as mean ± SEM

	Tumor	Liver	Spleen
Free ^{64}Cu, n=3	11 ± 3	14 ± 1	2 ± 0.6
^{64}Cu-DOTA, n = 3	1 ± 0.3	1 ± 0.2	0.3 ± 0.05
^{64}Cu-DOTA-3R-C18, n = 5	18 ± 8	10 ± 1	2 ± 0.07
LIP, n = 4	43 ± 31	9 ± 2	9 ± 5
